# A Modern Computer Application to Model Rare Earth Element Ion Behavior in Adsorptive Membranes and Materials

**DOI:** 10.3390/membranes13020175

**Published:** 2023-02-01

**Authors:** Aleksandra Rybak, Aurelia Rybak, Spas D. Kolev

**Affiliations:** 1 Faculty of Chemistry, Silesian University of Technology, Strzody 7, 44-100 Gliwice, Poland; 2 Faculty of Mining, Safety Engineering and Industrial Automation, Silesian University of Technology, 44-100 Gliwice, Poland; 3 School of Chemistry, The University of Melbourne, Melbourne, VIC 3010, Australia

**Keywords:** computer application, REE recovery, adsorptive materials, adsorption kinetics, REE behavior modelling, coal fly ashes

## Abstract

The following paper offers a modern REE 1.0 computer application designed to model the behavior of REE ions in adsorptive materials and membranes. The current version of the application is based on several models, such as the Lagergren pseudo-first order, pseudo-second-order and Elovich kinetic models, and the intraparticle diffusion model, the diffusion-chemisorption model, and the Boyd model. The application has been verified on a sample of four different types of adsorptive materials and membranes. The proposed application allowed the analysis of kinetics, but also the mechanisms of the adsorption process, especially those responsible for the rate-determining steps. It was found that Lagergren pseudo-second-order kinetic model was the best-fit model to describe the adsorption behavior of REE ions onto the novel materials and membranes. Other models determined the process of chemisorption was in force for the analyzed cases, and the mechanisms controlling the adsorption processes are diffusion-chemisorption and adsorption is mostly controlled by film diffusion. Additionally, characteristic parameters, such as q_e_ designated from two different models, showed very similar values, which indicates the correctness of the analysis.

## 1. Introduction

Nowadays, rare earth elements (REEs) become more and more important, especially because of their exceptional chemical, catalytic, physical, magnetic, and luminescent features and of course application in many modern technologies [1,2]. Due to China monopolizing in the production of REEs and the continuous increase in demand for these metals, there was a need to find new alternative sources and create new technologies for their recovery [2,3,4]. Unfortunately, the conventional REE mining techniques, which include ore deposits, are energy intensive and generate significant volumes of toxic wastes [5]. It turned out that coal fly ashes, generated annually worldwide in the amount of over 750 million tons, from which only 30% are utilized, can be their ideal source [1]. For REE recovery from coal fly ashes, physical, biological, and chemical methods (acid-base leaching) can be used [6,7,8,9,10,11,12,13,14,15,16,17,18]. However, they have many limitations. Therefore, alternative methods were introduced, such as membrane techniques and the application of appropriate materials, such as ion-imprinted polymers (IIP), which have recognition sites in a macromolecular matrix made by using a template molecule [19,20]. 

Currently, the most commonly used methods in water, municipal and industrial sewage purification, processing of extracts from chemical recovery methods, as well as the removal and recovery of them heavy metal ions are chemical precipitation, ion exchange, electrodialysis, ultrafiltration, nanofiltration, reverse osmosis, coagulation, flocculation, flotation, etc. [18,19,20,21,22,23,24]. However, they have several restrictions, such as a high consumption of reagents, generating toxic waste, and unpredictable removal of metal ions [25]. In turn, adsorption processes are by definition simple, economic, effective, and versatile. Of course, despite many advantages, they also have some disadvantages, such as low capacity, high costs, low selectivity, problems with their regeneration, and difficulties with scaling-up [26]. Therefore, further research is conducted towards the combination of advantages of usually used techniques, especially membrane techniques and adsorption processes, which was reflected in the introduction of a new type of adsorption membrane. As the name suggests, among the most important processes is adsorption, which is a mass transfer, during which the substance is transferred from the liquid phase to the surface of the solid and is associated with it as a result of physical or/and chemical interactions. As a rule, chemical adsorption is more suitable for removing or recovering metal ions, due to the stronger type of interaction and higher adsorption capacity relative to heavy metals. Special functional groups on the adsorbent surface that affect metal ions are responsible for this. That results in the adsorption separation of metals from the solution [27].

These special types of adsorption materials and membranes are a combination of functional groups (e.g., amino, carboxylic, and sulfone) with the surface and walls of pores of polymers [28,29,30,31,32,33,34,35,36]. They can also be hybrid materials and membranes that are a combination of polymer membranes with inorganic additives in the form of an adsorbent addition [37,38,39,40,41,42]. During the flow of processed sewage or extract through this type of membrane, functional active sites interact with separated ions, resulting in the separation of pollution or analytes at high speed and adsorption capacity. This is most likely caused by a very short intraparticle diffusion between the target substances and the active binding site in the adsorption membrane [28]. In recent years, the so-called ion-imprinted polymers (IIPs) are becoming more and more popular, due to their extremely high selectivity. IIPs are crosslinked polymers with pores and binding sites for the targeted ions [43,44,45]. They can be synthesized in a few steps during the reaction of the functional monomer, a crosslinker, an initiator and a template. In the first stage, complexes are formed based on monomers with functional groups and template ions (REE ions). In the second stage, polymerization of monomers is carried out by adding crosslinking agents and carrying out photo- or thermopolymerization. In the third step, template ions are removed from the polymers, thus creating specific binding sites that can later capture specific ions [46]. Ion-imprinted polymers are characterized by excellent ionic selectivity due to the presence of these specific sites that bind ions of the appropriate size and charge. Of course, their adsorption capacity depends on many factors, such as the ability of IIPs ligands to bind metal ions, the size of the ions, their charge, the electron configuration of the metals, and the degree of oxidation [47,48]. These types of materials are characterized by appropriate thermal and pH stability [49]. IIPs are developed to mimic the key and lock mechanisms for recognizing and removing target ions. Thus, IIPs are characterized by excellent selectivity and show a specific affinity for a given ion. Even pollutants or analytes present in low concentrations can be selectively removed by IIPs, which has not been successfully achieved by other methods [50]. A very important issue, detailed in the case of analyzing the received data from the course of experience using adsorption materials and membranes, is their proper interpretation and the answer to the question with what kind of adsorption we are dealing with and what types of mechanisms are responsible for separating processes. Therefore, in order to analyze process kinetics, Lagergren (Lagergren Pseudo-First-order and Pseudo-Second-order Kinetic Models) and Elovich models are used. However, to analyze the adsorption mechanisms, intraparticle diffusion, diffusion-chemisorption, and Boyd models were used [41]. Usually, researchers dealing with the analysis of metal ion adsorption processes use popular models for the interpretation of experimental data, without delving into the mechanisms responsible for these processes. As a result, there is no suitable comprehensive tool that would fulfill both functions. In addition, it is known that only such information on the mechanisms of the considered processes will enable setting the direction for further research and introducing appropriate modifications of the analyzed adsorbents, which will allow obtaining materials with improved adsorption properties.

The aim of this work is the creation of a novel computer application, REE 1.0, to model REE ion behavior in adsorptive materials and membranes to characterize the obtained experimental results and eventually to select the most optimal materials. The proposed application will be appropriate both for the analysis of kinetics and the mechanisms responsible for separation processes in adsorption materials. It will also enable determining characteristic parameters for adsorption and diffusion processes. The REE 1.0 application will be an ideal tool for researchers studying the adsorption processes of metal ions, not only REE but on various types of adsorbents. They will be able to find a full list of popular models designed to characterize them, as well as less frequently used models that provide valuable data on both the kinetics of processes and the mechanisms governing them. The proposed application is an initial version, which will be appropriately modified over time to a more advanced form.

## 2. Materials and Methods

The created application is based on various models, which are given below.

### 2.1. Lagergren Kinetic Models

Lagergren pseudo-first-order and pseudo-second-order kinetic models can be linearly expressed as Equations (1) and (2), respectively [51,52,53]:

ln (𝑞_𝑒_ − 𝑞_𝑡_) = ln 𝑞_𝑒_ − 𝑘_1_𝑡, (1)

𝑡/𝑞_𝑡_ = 1/𝑘_2_𝑞^2^𝑒 + 𝑡/𝑞_𝑒_, (2)

where 

𝑘_1_ (min^−1^)—the rate constant of the pseudo-first-order kinetic model, 

𝑘_2_ (gmg^−1^ min^−1^)—the rate constant of the pseudo-second-order kinetic model, 

𝑞_𝑡_—the adsorption capacity of metal ions at time 𝑡 (min), and

𝑞_𝑒_ (mg/g)—the adsorption capacity of metal ions at equilibrium state.

Lagergren kinetic equations could be a helpful tool to assess adsorbent adsorption performance.

### 2.2. The Elovich Model

Usually, the Elovich model was used in the analysis of chemisorption kinetics of gases on solid surfaces. However, according to Wang et al. [54], it could be also used in the investigation of liquid state sorption of an adsorbent and could be expressed using the following equation [54,55]:

𝑞_𝑡_ = 𝑎 + 𝑏ln (𝑡), (3)

where 𝑎 (mg/g) and 𝑏 are the Elovich parameters. These parameters could be obtained from the intercept and slope of the created straight line.

### 2.3. Mechanism Insights

To explain the adsorption behaviors of metal ions, it is important to gain insight into the adsorption mechanism, using the intraparticle diffusion model, the diffusion chemisorption model and the Boyd equation [56,57,58,59].

#### 2.3.1. Intraparticle Diffusion

The intraparticle diffusion model [56,57] can be expressed using the following equation:

𝑞_𝑡_ = 𝑥_𝑖_ + 𝑘_𝑝_𝑡 ^0.5^ (4)

where

*q_t_* [mg/g]—the adsorbed amount at time *t* [min],

*k_p_* [mg g^−1^min^−1/2^]—the intraparticle diffusion rate constant, and

*x_i_* [mg/g]—the intercept of straight-line *q_t_* (*t* ^0.5^), related to the boundary layer thickness.

It turns out that if the plot *q_t_* (*t* ^0.5^) is a straight line, the adsorption is controlled by the intraparticle diffusion. However, if we have to use multilinear curves, then two or more mechanisms influence the adsorption process. 

#### 2.3.2. The Diffusion-Chemisorption Model

This model can be expressed as the linear relationship [58]:

𝑡 ^0.5^/𝑞_𝑡_ = 1/𝐾_DC_ + 1/𝑞_𝑒_ 𝑡 ^0.5^ (5)

where *K_DC_* is the diffusion-chemisorption constant.

#### 2.3.3. The Boyd Equation

This type of equation could be used to determine the rate controlling step during the adsorption [58,59,60,61]:

𝐹 = 1 − (6/𝜋^2^) exp (−𝐵𝑡) (6)

where 

*Bt*—a function of *F*, which is the fraction of solute adsorbed at different times. *F* values could be obtained using equation:

*F* = *q_t_*/*q_e_* (7)

where

*q_t_* [mg/g]—the adsorbed amount at time t [min], and

𝑞_𝑒_ [mg/g]—the adsorbed amount of metal ions at equilibrium state.

The *Bt* values at different contact times could be calculated using the following formula (if *F* > 0.85) [3]:

*Bt*= −0.4977 − ln (1 − *F*) (8)

The plot of the Boyd model can be obtained using the relationship between *Bt* versus time *t*.

Based on this model, the effective diffusion coefficient can be calculated by usage of the following equation:

ln [1/(1 − *F*^2^)] = *π*^2^*/r*^2^
*D_e_t* (9)

Creating the plot of ln [1/(1 − *F*^2^(*t*))] versus time *t*, the diffusion coefficient *D_e_* can be calculated from the slope *π*^2^*D_e_/r*^2^.

It is stated that if the chart *Bt*(*t*) is a straight line and passes through the beginning of the coordinate system, then the process controlling the rate of the mass transport will be pore diffusion (or particle diffusion mechanism). Otherwise, if the *Bt*(*t*) dependence chart is non-linear or linear, but it does not pass through the origin, the film-diffusion or external mass transport will be the main dominant factors.

### 2.4. Analysed Materials

The operation of the proposed computer application was verified on the basis of sample experimental data of the adsorption process of selected REE ions on various types of adsorption materials. For this purpose, experimental data presented in 4 different publications were used [62,63,64,65]. The first type was Ce (III) ion imprinted materials based on 2-hydroxyethyl methacrylate (HEMA) and N-methacryloylamido antipyrine (MAAP) as functional monomers [62]. The second analyzed material was synthesized as a Lu (III)-ion-imprinted polymer, based on Lu (III)-4-vinylpyridine-acetylacetone complex as a functional monomer and ethylene glycol dimethacrylate as a crosslinker [63]. The third type of adsorption material has been synthesized by Liu et al. [64] in the form of diglycolamide polymer-grafted silica. The last type of adsorbents [65] were the imprinted mesoporous cellulose nanocrystals films (IMCFs).

## 3. Results and Discussion

A Novel Computer Application, REE 1.0, for Modeling of REE Ion Behavior in Adsorptive Materials

In the initial stage of research, the authors created a computer program REE 1.0 in the Java programming language. In this program, the user will be able to choose the appropriate kinetic model and adsorption mechanism model for the adsorptive materials and membranes, such as the Lagergren pseudo-first order, pseudo-second-order and Elovich kinetic models and the intraparticle diffusion model, the diffusion-chemisorption model, and the Boyd model to study the various mechanisms. In this work, the experimental results found throughout the literature [62,63,64,65] were compared with the theoretical data predicted by means of the mentioned models. The user could enter the parameters, such as the adsorption capacity (*q_t_*) of REE ions at contact time (*t*). The program calculates the values of characteristic parameters, such as the adsorption capacity of metal ions at equilibrium state *q_e_*, rate constant *k*_1_ and *k*_2_, the intraparticle rate constant *k_p_* and boundary layer thickness *x_i_*, the diffusion-chemisorption constant *K_DC_*, effective coefficient *D_e_* and determination coefficient *R*^2^. There is also the possibility to compare the experimental results with theoretical one. It will be an excellent tool for research scientists to study the adsorption kinetics and various mechanisms responsible for the adsorption of REE ions (and other metal ions) on the adsorptive membranes and other adsorptive materials.

**Figure 1:**
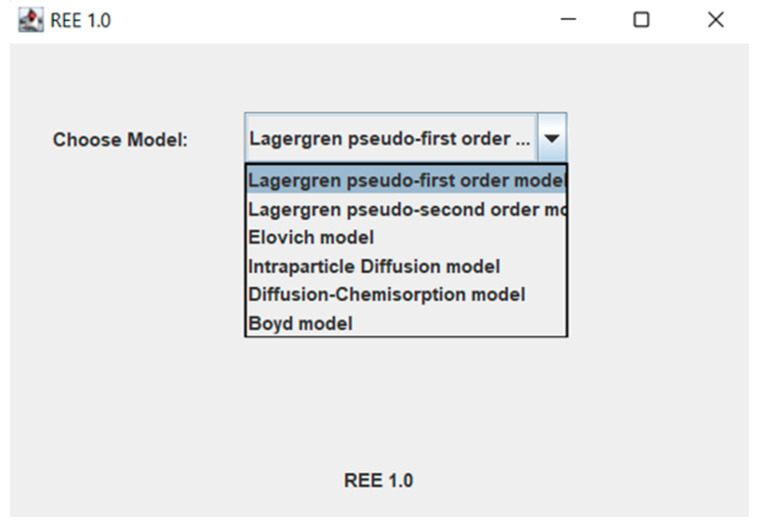
Main window for kinetic model or adsorption mechanism model selection.

**Figure 2:**
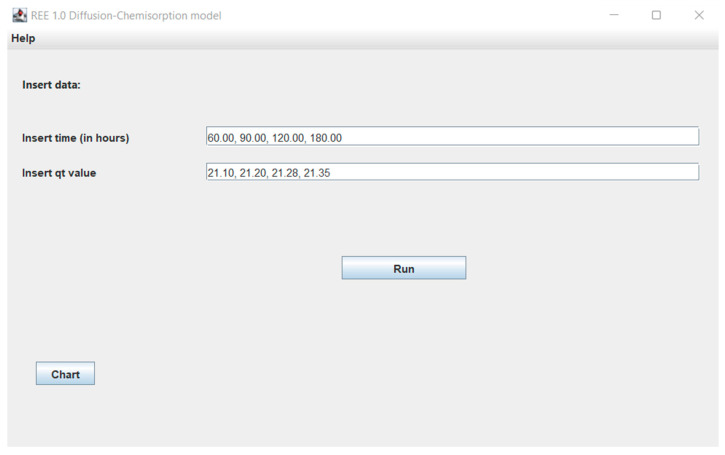
Window with diffusion-chemisorption model experimental data results for Eu ions.

**Figure 3:**
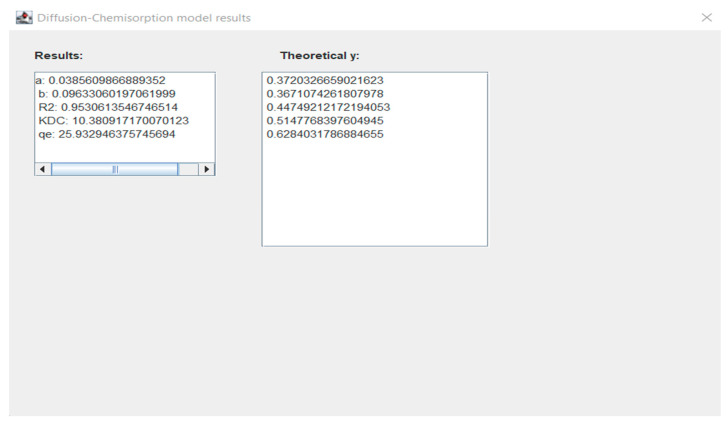
Window with diffusion-chemisorption model results for Eu ions.

**Figure 4:**
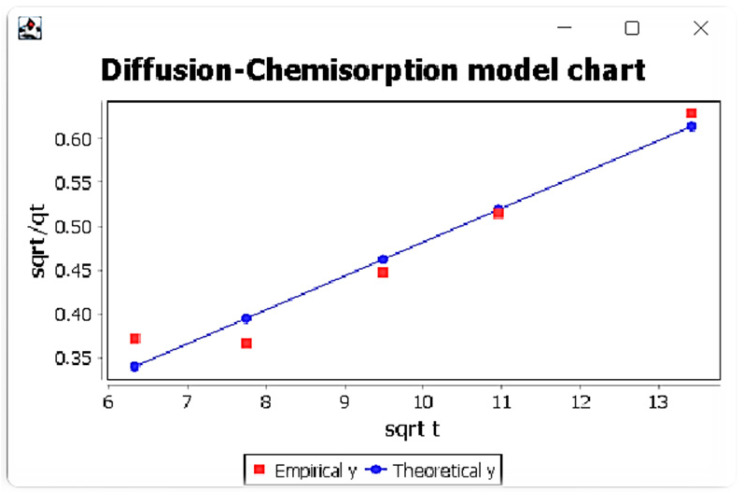
Window with diffusion-chemisorption model results and comparison of experimental and theoretical data graph for Eu ions.

The program consists of the following windows:

- Main window for model selection (Figure 1). Individual models are selected from the combo box.

- In the next windows (Figure 2), additional tools such as the ability to add user’s experimental data, theoretical results from various models (Figure 3), the possibility to present the theoretical and experimental data at the graphs (Figure 4) are available.

To compare the theoretical results obtained from the REE 1.0 application based on various models with the experimental results obtained from the literature, the correlation coefficient *R*^2^ was calculated. The data obtained during the simulation using REE 1.0 application for various models were presented in Table 1.

As can be seen from Table 1, adsorptive materials and membranes are successfully used in the separation of rare earth metal ions from various types of aquatic solutions.

Kecili et al. [62] have examined the novel Ce (III) ion imprinted materials based on 2-hydroxyethyl methacrylate (HEMA) and N-methacryloylamido antipyrine (MAAP) as functional monomers. These materials were used for separation of Ce (III) ions from aquatic solutions and mixtures with other lanthanide ions, such as La (III) and Nd (III). However, the authors were only interested in examination of equilibrium parameters of adsorption process, using the Langmuir and Freundlich models. They have found that binding of Ce (III) ions to the ion imprinted poly (HEMA-co-(MAAP)_2_-Ce(H_2_O)_2_) is well fitted to the Langmuir isotherm model. It means that the binding of Ce (III) ions to the ion imprinted poly (HEMA-co-(MAAP)_2_Ce(H_2_O)_2_) is monolayer. They have also found that obtained IIPs exhibit high selectivity and sensitivity towards Ce (III) ions. In turn, Lai et al. [63] have synthesized a Lu (III)-ion-imprinted polymer, based on Lu (III)-4-vinylpyridine-acetylacetone complex as a functional monomer and ethylene glycol dimethacrylate as a crosslinker. Additionally, again, the authors have only used the Langmuir and Freundlich models and found that the Lu (III) ion adsorption was fitted to the Langmuir model, indicating that they have to use a monolayer adsorption of Lu (III) ions. The synthesized Lu (III) IIP had the properties of a large surface area, high adsorption capacity, a small cavity, a fast adsorption rate, and favorable heat stability. The results showed that the maximum adsorption of Lu (III) IIP was 64.2 mg g^−1^ with an adsorption equilibrium time of 30 min and the optimum pH was 5.5. The synthesized Lu (III) IIP had a good selective recognition ability for Lu (III) ion, compared with other ions. Liu et al. [64] have synthesized the new materials for REE ion adsorption, based on diglycolamide polymer-grafted silica. The prepared materials were used for separation of Eu (III) ions from solutions with other metal ions, such as K(I), Cr (II), Cu (II) or Fe (III). It was found that their occurrence did not decrease the adsorption of REE ion, so these materials had a good selective recognition ability for Eu (III) ion. In order to determine the equilibrium relationship between the adsorbent and the adsorbate, the authors have used the Freundlich and Langmuir isotherms. With their help, the ratio of the amount of adsorbed substance to its amount remaining in the solution (at a fixed temperature and in a state of equilibrium) and the sorption capacity of the adsorbent were determine. They found that the Langmuir adsorption isotherm was suited better to experimental data, which means the occurrence of monolayer adsorption, the energy equivalence of adsorption places and the lack of interactions between molecules adsorbed on adjacent active sites. In addition, all active places on the sorbent are free sites, ready to accept sorbate from the solution. In turn, using Lagergren models, the authors analyzed the kinetics of adsorption, and in particular the possible rate-determining step of the adsorption process. The experimental results were found to better fit the pseudo-second-order kinetic model. Further, the experimental *q_e,exp_* values agree well with the theoretical *q_e,theor_* for the pseudo-second-order kinetic model. This indicates that surface chemical sorption may be the step determining the rate of Eu (III) ion adsorption on analyzed IIP. Zheng et al. [65] have synthesized the imprinted mesoporous cellulose nanocrystals films (IMCFs) and used them for Nd (III) ions separation from aqueous solutions. For the analysis of experimental data, the Lagergren, Langmuir and Freundlich models were used. The authors have found that the Lagergren pseudo-second-order model fitted well the experimental data and the analyzed adsorption process belongs to chemical adsorption. In terms of the adsorption performance of IMCFs on Nd (III), again, the authors found a better fit of experimental results to the Langmuir isotherm, which proves monolayer sorption.

Analyzing the experimental results published in articles [62,63,64,65] on the adsorption of various rare earth metal ions from aqueous solutions, most often with accompanying ions (other REE and matrix ions) using the developed REE 1.0 application, the following conclusions were drawn. Of course, the results partly coincided with the conclusions of the authors using the Lagergren model, but they were supplemented with important information from the rest of the models used in the application.

It was found that in all cases, the experimental data from the adsorption analysis showed a better fit to the pseudo-second-order Lagergren model, which indicates the presence of chemical sorption. This was found based on higher *R*^2^ values compared to the pseudo-first-order model. In addition, the differences between the values of *q_e_* determined during the simulation and *q_e_* obtained experimentally were significantly smaller for the pseudo-second-order Lagergren model. In addition, it was also found that the experimental results fit the Elovich model, which proves the occurrence of chemisorption. It is also very important to determine what type of mechanism is responsible for the sorption and separation processes, and in particular which of them is responsible for the rate of these processes. The following models are used for this: intraparticle diffusion, diffusion-chemisorption, and Boyd. In all analyzed cases, a characteristic course of the relationship *q_t_* (*t*^0.5^) was found. Namely, they were in the form of multilinear curves (the value of *R*^2^ was sufficiently low), which proves the influence of two or more mechanisms on the adsorption process, and not only intraparticle diffusion. Using this model, it is possible to determine characteristic parameters, such as the intraparticle diffusion rate constant and the boundary layer thickness. It was found that the highest parameter values were obtained for Lu (III)IIP, and the lowest for Ce (III)IIP and Nd (III)IIPs. This is directly related to the form of adsorption materials.

It was also found that the diffusion-chemisorption mechanism was the dominant mechanism, taking into account its highest values of correlation coefficients among the considered models and the linear course of the relationship *t*^0.5^*/q_t_* (*t*^0.5^). This model also allowed to determine two parameters, namely the adsorbed amount of metal ions at equilibrium state *q_e_* and diffusion-chemisorption constant *K_DC_*. The highest values of these parameters were obtained for Lu (III)IIPs. In addition, it was found that the values of adsorbed amount of metal ions at equilibrium state *q_e_* are very close to their values determined from the pseudo-second-order Lagergren model.

Then, moving on to the model based on the Boyd equation, it was found that for Ce (III) and Eu (III), the relationship 𝐵𝑡(*t*) is non-linear, while for Lu (III) and Nd (III) despite the linear relationship, the lines do not pass through the origin, which proves that film-diffusion or external mass transport will be the main dominant factors. The calculated values of *D_e_* fall well within the values characteristic for chemisorption systems.

Thus, we can see that the use of a computer application with an extensive range of models, concerning not only the analysis of kinetics, but also the mechanisms governing the adsorption processes of REE ions, both in modern materials and adsorption membranes, gives a number of possibilities, along with determining the characteristic parameters of the described processes.

## 4. Conclusions

In this paper, the authors proposed a modern computer application, REE 1.0, to model REE ion behavior in adsorptive membranes and materials. The current version of application was based on a few models, such as the Lagergren pseudo-first-order and pseudo-second-order and Elovich kinetic models and the intraparticle diffusion model, the diffusion-chemisorption model, and the Boyd model to study the various adsorption mechanisms. The operation of this application has been verified based on experimental results from the literature, regarding REE ion adsorption on four different types of adsorptive materials and membranes. It was found that the pseudo-second-order kinetic model was the best-fit model to describe the adsorption behavior of REE ions onto the novel materials and membranes and that the adsorption mechanism was a chemical coordination process. The good fit of experimental results to the Elovich model (*R*^2^ > 0.83) also indicates the existence of chemisorption. In the case of the intraparticle model, the linear fit exhibited multilinear curves (*R*^2^ < 0.84), so two or more steps will influence the adsorption process. In turn, the good fit to the diffusion-chemisorption model (*R*^2^ > 0.95) and the similarity of 𝑞_𝑒_ values with those obtained via the Lagergren pseudo-second-order kinetic model indicate that the adsorption of REE ions on the examined materials can be described using this model. The Boyd equation provided additional data, namely it was found that none of the obtained graphs passed through the origin. This indicated that the adsorption is mostly controlled by film diffusion. Additionally, the calculated values of *D_e_* fall well within the values characteristic for chemisorption systems.

The created application, REE 1.0, could be an excellent tool for scientists studying adsorptive materials and membranes. REE 1.0 enables not only analyzing the kinetic mechanisms, but above all to studying the mechanisms responsible for the adsorption and behavior of REE ions in these types of materials and membranes. The proposed application is a preliminary version of the program and, during further research, it will be appropriately modified, especially as regards the number of available adsorption models and supplemented with elements regarding the type of adsorbents and their characteristics.

## Figures and Tables

**Table 1:** Comparison of simulation results for various REE ions obtained in REE 1.0 application.

REE Ion	Material	Model	*q_e_* [mg/g]	*K*_1_ [min^−1^]	*K*_2_ [gmg^−1^min^−1^]	*a*	*b*	*K_p_* [mgg^−1^ min^−1/2^]	*x_i_* [mg/g]	*K_DC_*	*D_e_* [m^2^/min]	*R* ^2^	Ref.
Ce	Ce(III) (HEMA-co-(MAAP)_2_Ce(H_2_O)_2_)	I pseudo-order	1.59	0.03								0.73	[62]
		II pseudo-order	6.92		0.02							0.99	
		Elovich				1.02	1.92					0.83	
		Intraparticle						0.27	3.87			0.69	
		Diffusion-chemisorption	7.62							4.23		0.98	
		Boyd									1.66 × 10^−17^	0.74	
Lu	Lu(III)–4-vinylpyridine–acetylacetone-EDMA	I pseudo-order	42.71	0.04								0.90	[63]
		II pseudo-order	74.63		0.002							0.99	
		Elovich				15.69	9.87					0.97	
		Intraparticle						10.82	6.7			0.69	
		Diffusion-chemisorption	76.33							62.11		0.98	
		Boyd									4.23 × 10^−17^	0.90	
Eu	diglycolamide polymer modified silica	I pseudo-order	18.90	0.02								0.96	[64]
		II pseudo-order	23.50		0.04							0.99	
		Elovich				0.23	20.16					0.99	
		Intraparticle						1.69	3.33			0.84	
		Diffusion-chemisorption	25.93							10.38		0.95	
		Boyd									6.49 × 10^−18^	0.68	
Nd	imprinted mesoporous cellulose nanocrystals films (IMCFs)	I pseudo-order	16.95	0.004								0.87	[65]
		II pseudo-order	25.00		0.0002							0.99	
		Elovich				4.25	12.09					0.98	
		Intraparticle						1.69	3.33			0.80	
		Diffusion-chemisorption	28.09							1.46		0.97	
		Boyd									9.13 × 10^−19^	0.94	

## Data Availability

The data presented in this study are available on request from the corresponding author. The data are not publicly available due to the extremely large size.
